# Modelling COVID-19 vaccination status and adherence to public health and social measures, Eastern Mediterranean Region and Algeria

**DOI:** 10.2471/BLT.22.288655

**Published:** 2022-12-01

**Authors:** Zlatko Nikoloski, Robert Bain, Manal K Elzalabany, Peggy Hanna, Tara Rose Aynsley, Dalia Samhouri, Leonardo Menchini, Neha Kapil, Amaya Gillespie

**Affiliations:** a Department of Health Policy, London School of Economics and Political Science, Houghton Street, London WC2A 2AE, England.; b United Nations Children’s Fund Regional Office for the Middle East and North Africa, Amman, Jordan.; c World Health Organization Regional Office for the Eastern Mediterranean, Cairo, Egypt.

## Abstract

**Objective:**

To study the link between coronavirus disease 2019 (COVID-19) vaccination status and adherence to public health and social measures in Members of the Eastern Mediterranean Region and Algeria.

**Methods:**

We analysed two rounds of a large, cross-country, repeated cross-sectional mobile phone survey in June–July 2021 and October–November 2021. The rounds included 14 287 and 14 131 respondents, respectively, from 23 countries and territories. Questions covered knowledge, attitudes and practices around COVID-19, and demographic, employment, health and vaccination status. We used logit modelling to analyse the link between self-reported vaccination status and individuals’ practice of mask wearing, physical distancing and handwashing. We used propensity score matching as a robustness check.

**Findings:**

Overall, vaccinated respondents (8766 respondents in round 2) were significantly more likely to adhere to preventive measures than those who were unvaccinated (5297 respondents in round 2). Odds ratios were 1.5 (95% confidence interval, CI: 1.3–1.8) for mask wearing; 1.5 (95% CI: 1.3–1.7) for physical distancing; and 1.2 (95% CI: 1.0–1.4) for handwashing. Similar results were found on analysing subsamples of low- and middle-income countries. However, in high-income countries, where vaccination coverage is high, there was no significant link between vaccination and preventive practices. The association between vaccination status and adherence to public health advice was sustained over time, even though self-reported vaccination coverage tripled over 5 months (19.4% to 62.3%; weighted percentages).

**Conclusion:**

Individuals vaccinated against COVID-19 maintained their adherence to preventive health measures. Nevertheless, reinforcement of public health messages is important for the public’s continued compliance with preventive measures.

## Résumé

**Objectif:**

Étudier le lien entre le statut de vaccination contre la maladie à coronavirus 2019 (COVID-19) et l'adhésion aux mesures sociales et de santé publique en Algérie et au sein des Membres de la Région de la Méditerranée orientale.

**Méthodes:**

Nous avons analysé deux séries de résultats issus d'une vaste enquête transnationale et transversale réalisée à intervalles réguliers via téléphone mobile en juin–juillet 2021 et octobre–novembre 2021. Ces séries ont comptabilisé respectivement 14 287 et 14 131 participants répartis sur 23 pays et territoires. Les questions portaient sur les connaissances, attitudes et pratiques relatives à la COVID-19, ainsi que sur le statut démographique, professionnel, sanitaire et vaccinal. Nous avons utilisé un modèle Logit pour examiner le lien entre le statut vaccinal rapporté et les pratiques de chacun en matière de port du masque, de distanciation physique et de lavage des mains. Enfin, nous avons procédé à un appariement des coefficients de propension en guise de test de robustesse.

**Résultats:**

Globalement, les répondants vaccinés (8766 dans la deuxième série) étaient nettement plus enclins à respecter les mesures de prévention que les répondants non vaccinés (5297 dans la deuxième série). L'odds ratio s'élevait à 1,5 (intervalle de confiance de 95%, IC: 1,3–1,8) pour le port du masque; 1,5 (IC de 95%: 1,3–1,7) pour la distanciation physique; et 1,2 (IC de 95%: 1,0–1,4) pour le lavage des mains. Des résultats similaires ont été obtenus lors de l'analyse des sous-échantillons provenant de pays à revenu faible et intermédiaire. Cependant, dans les pays à revenu élevé où la couverture vaccinale est importante, aucune corrélation n'a été établie entre la vaccination et les pratiques préventives. Le lien entre le statut vaccinal et l'adhésion aux mesures de santé publique s'est maintenu au fil du temps, même si la couverture vaccinale a triplé en cinq mois (passant de 19,4% à 62,3%; pourcentages pondérés).

**Conclusion:**

Les personnes vaccinées contre la COVID-19 ont continué à respecter les mesures préventives en matière de santé. Il faut toutefois renforcer les messages de santé publique afin que la population ne renonce pas à appliquer ces mesures.

## Resumen

**Objetivo:**

Analizar la relación entre el estado de vacunación contra la enfermedad por coronavirus de 2019 (COVID-19) y el cumplimiento de las medidas sociales y de salud pública en los Estados Miembros de la Región del Mediterráneo Oriental y Argelia.

**Métodos:**

Se analizaron dos rondas de una gran encuesta transversal de telefonía móvil repetida entre países realizada en junio y julio de 2021 y en octubre y noviembre de 2021. Las rondas incluyeron 14 287 y 14 131 encuestados, respectivamente, de 23 países y territorios. Las preguntas incluían conocimientos, actitudes y prácticas en relación con la COVID-19, así como la situación demográfica, laboral, sanitaria y de vacunación. Se utilizó un modelo logit para analizar la relación entre el estado de vacunación informado por los encuestados y la práctica del uso de mascarillas, el distanciamiento físico y el lavado de manos. Se utilizó el emparejamiento por puntuación de propensión como comprobación de consistencia.

**Resultados:**

En general, los encuestados vacunados (8766 encuestados en la ronda 2) eran significativamente más propensos a cumplir las medidas preventivas que los no vacunados (5297 encuestados en la ronda 2). Las razones de posibilidades fueron de 1,5 (intervalo de confianza del 95 %, IC: 1,3-1,8) para el uso de mascarilla; 1,5 (IC del 95 %: 1,3-1,7) para el distanciamiento físico; y 1,2 (IC del 95 %: 1,0-1,4) para el lavado de manos. Se encontraron resultados similares al analizar submuestras de países de ingresos bajos y medios. Sin embargo, en los países de ingresos altos, donde la cobertura de vacunación es alta, no hubo una relación significativa entre la vacunación y las prácticas preventivas. La asociación entre el estado de vacunación y el cumplimiento de las recomendaciones de salud pública se mantuvo en el tiempo, a pesar de que la cobertura de vacunación informada por las personas se triplicó en 5 meses (del 19,4 % al 62,3 %; porcentajes ponderados).

**Conclusión:**

Las personas vacunadas contra la COVID-19 mantuvieron su adhesión a las medidas sanitarias preventivas. No obstante, el refuerzo de los mensajes de salud pública es importante para que la población siga cumpliendo las medidas preventivas.

## ملخص

**الغرض:**

دراسة الصلة بين حالة التطعيم ضد مرض فيروس كورونا 2019 (كوفيد 19)، والالتزام بالصحة العامة والتدابير الاجتماعية لدى أعضاء منطقة شرق البحر المتوسط والجزائر.

**الطريقة:**

قمنا بتحليل جولتين من مسح واسع مقطعي ومتكرر، يشمل عدة دول عبر الهاتف المحمول في يونيو/حزيران - يوليو/تموز 2021، وأكتوبر/تشرين أول - نوفمبر/تشرين ثاني 2021. وشملت الجولتان 14287 و14131 مستجيبًا على الترتيب من 23 دولة وإقليم. غطت الأسئلة المعرفة، والمواقف، والممارسات الخاصة بكوفيد 19، والحالة السكانية، والتوظيف، والصحية، والتطعيم. استخدمنا وضع النماذج اللوغاريتمية لتحليل الصلة بين حالة التطعيم المبلغ عنها ذاتيًا، وممارسة الأفراد لارتداء الأقنعة، والتباعد الجسدي، وغسل اليدين. استخدمنا مطابقة درجة النزوع كفحص للفعالية.

**النتائج:**

بشكل عام، كان المستجيبون الذين تم تطعيمهم (8766 مستجيبًا في الجولة الثانية) أكثر ميلًا للالتزام بالتدابير الوقائية من أولئك الذين لم يتم تطعيمهم (5297 مستجيبًا في الجولة الثانية). كانت نسب الاحتمالات 1.5 (بفاصل ثقة مقداره 95%: 1.3 إلى 1.8) لارتداء الأقنعة؛ و1.5 (بفاصل ثقة مقداره 95‏%: 1.3 إلى 1.7) للتباعد الجسدي؛ و1.2 (بفاصل ثقة مقداره 95‏%: 1.0 إلى 1.4) لغسل اليدين. تم العثور على نتائج مماثلة في تحليل لعينات فرعية من الدول منخفضة الدخل والدول متوسطة الدخل. ومع ذلك، فإنه في الدول ذات الدخل المرتفع، حيث تكون تغطية التطعيم عالية، لم يكن هناك صلة ملموسة بين التطعيم والممارسات الوقائية. استمر الارتباط بين حالة التطعيم والالتزام بنصائح الصحة العامة عبر الوقت، على الرغم من أن تغطية التطعيم المبلغ عنها ذاتيًا تضاعفت ثلاث مرات خلال 5 أشهر (19.4% إلى 62.3%؛ النسب المئوية المرجحة).

**الاستنتاج:**

حافظ الأفراد الذين تم تطعيمهم ضد كوفيد 19 على التزامهم بالتدابير الصحية الوقائية. ورغم ذلك، فإن تعزيز رسائل الصحة العامة مهم لاستمرار التزام العامة بالتدابير الوقائية.

## 摘要

**目的:**

旨在研究东地中海地区成员国和阿尔及利亚新型冠状病毒肺炎（新冠肺炎）疫苗接种状况与公共卫生和社会措施遵从情况之间的关联性。

**方法:**

我们分析了 2021 年 6-7 月和 2021 年 10-11 月重复开展的两轮具有代表性的大型跨国性横断面手机调查。这两轮调查分别邀请了来自 23 个国家和地区的 14,287 和 14,131 名受访者参与。问题涵盖对新型冠状病毒肺炎的了解、态度和应对方法，以及人口、就业、健康和疫苗接种状况。我们使用分类评定模型进行建模，以分析自报疫苗接种状况与个人佩戴口罩、保持物理距离和洗手等行为之间的关联性。我们使用倾向评分匹配方法作为稳健性检验手段。

**结果:**

总体而言，接种疫苗的受访者（第 2 轮中的 8,766 名受访者）明显比未接种疫苗的受访者（第 2 轮中的 5,297 名受访者）更有可能遵从预防措施。各项措施的优势比分别为：佩戴口罩为 1.5（95％ 置信区间，CI：1.3-1.8)；保持物理距离为 1.5 (95% CI: 1.3-1.7)；以及洗手为 1.2 (95% CI: 1.0–1.4)。在分析中低收入国家的子样本时也得出了类似的结果。但是，在疫苗接种率较高的高收入国家，疫苗接种和遵从预防措施之间并无明显关联。虽然自报疫苗接种覆盖率在 5 个月内增加了两倍（19.4% 至 62.3%；权重比例），但是疫苗接种状况与遵从公共卫生建议之间的关联性随着时间推移仍然持续存在。

**结论:**

接种新型冠状病毒肺炎疫苗的民众始终坚持遵从预防性健康措施。尽管如此，加强宣传公共卫生信息对于公众继续遵从预防措施仍然至关重要。

## Резюме

**Цель:**

Изучить связь между статусом вакцинации против коронавирусной инфекции 2019 г. (COVID-19) и соблюдением мер общественного здравоохранения и социальных мер в странах Восточного Средиземноморья и Алжире.

**Методы:**

Проведен анализ двух раундов крупного повторного сквозного опроса жителей разных стран по мобильным телефонам в июне-июле и октябре-ноябре 2021 года. В раундах приняли участие 14 287 и 14 131 респондент соответственно из 23 стран и территорий. Вопросы касались знаний, отношения и практических действий в отношении COVID-19, а также демографических данных, занятости, состояния здоровья и статуса вакцинации. Для анализа связи между собранными со слов пациента данными о статусе вакцинации и практике ношения маски, социальном дистанцировании и мытье рук авторы использовали logit-моделирование. Для проверки устойчивости модельного предположения авторы использовали метод отбора подобного по коэффициенту склонности.

**Результаты:**

В целом вакцинированные респонденты (8766 респондентов во 2-м раунде) значительно чаще придерживались профилактических мер по сравнению с невакцинированными (5297 респондентов во 2-м раунде). Отношение шансов составило 1,5 (95%-й ДИ: 1,3–1,8) для ношения маски, 1,5 (95%-й ДИ: 1,3–1,7) для социального дистанцирования и 1,2 (95%-й ДИ: 1,0–1,4) для мытья рук. Аналогичные результаты были получены при анализе подвыборок стран с низким и средним уровнем дохода. Однако в странах с высоким уровнем дохода, где охват вакцинацией высок, не было обнаружено существенной связи между вакцинацией и профилактическими мерами. Связь между статусом вакцинации и соблюдением рекомендаций по охране общественного здравоохранения сохранялась в течение долгого времени, даже несмотря на то, что за 5 месяцев охват вакцинацией, по данным, сообщенным пациентами, увеличился в три раза (с 19,4 до 62,3%; взвешенные процентные доли).

**Вывод:**

Лица, вакцинированные против COVID-19, продолжали соблюдать профилактические меры по охране здоровья. Тем не менее распространение информации об охране здоровья населения необходимо для дальнейшего соблюдения населением профилактических мер.

## Introduction

The introduction of vaccines for coronavirus disease 2019 (COVID-19) added another measure to the existing set of recommended preventive measures (wearing a mask in public, keeping a distance from other people and regular handwashing). The roll-out of the vaccines, however, raised concerns that vaccination may lead to lower adherence to the existing preventive measures. The advice from the World Health Organization (WHO) was to continue these public health and social measures after being vaccinated.^1^ However, evidence from other epidemics suggests that there is lower adherence to preventive measures when some level of protection exists (for example, individuals who use human immunodeficiency virus pre-exposure prophylaxis).^2^ This effect is further compounded by people losing motivation to follow recommended protective measures (so-called pandemic fatigue).^3^ With most countries relaxing the stringent restrictions imposed at the start of the pandemic, understanding the link between vaccination status and adherence to public health advice is important.

To date, a few studies from high-income countries have tried to understand the link between vaccination status and COVID-19 preventive behaviours. Relying on a longitudinal survey of vaccinated and unvaccinated individuals, a study from the United Kingdom of Great Britain and Northern Ireland^4^ found no evidence that vaccinated individuals decreased compliance relative to those who were not yet vaccinated. These findings were echoed in a study relying on a cross-sectional survey in 12 high-income countries.^5^

The Eastern Mediterranean Region (as defined by WHO) and the Middle East and North Africa Region (as defined by the United Nations Children’s Fund, UNICEF) together comprise an overlapping group of 23 countries and territories. Among these countries, only Algeria is not a Member of the WHO Eastern Mediterranean Regional Committee, but the WHO African Regional Committee. While vaccination uptake and barriers to uptake in these countries received much attention in 2021,^6^ the link between vaccination status and adherence to public health advice on COVID-19 prevention has not been sufficiently studied. Only one study, in Somalia, found a positive correlation between adherence to preventive behaviours and willingness to get vaccinated.^7^ However, there is little insight into what happens to COVID-19 preventive behaviours as vaccination rates across the region increase. We have identified 11 studies documenting the practice of the most common types of COVID-19 preventive behaviours in Egypt,^8^^,^^9^ the Islamic Republic of Iran,^10^^,^^11^ West Bank and Gaza Strip,^12^ Saudi Arabia^13^^–^^16^ and Somalia.^7^^,^^17^ The studies covered the general population,^7^^,^^9^^–^^13^^,^^15^^,^^17^ or medical professionals and medical students.^8^^,^^14^^,^^16^ There are a few common characteristics across these studies: (i) they were small-scale, cross-sectional surveys; (ii) except for the two studies in Islamic Republic of Iran, the available studies in the region relied on online data collection;^18^ and (iii) all of the studies pre-dated the main vaccination campaigns in the region.

Against this background, our objective was to study the relationship between COVID-19 vaccination status and adherence to public health and social measures throughout the Eastern Mediterranean and Middle East and North Africa. To account for the differences in vaccine availability across different countries, we also conducted a subregional analysis of countries by income group.

## Methods

### Setting

The analysis in this paper is based on a repeated cross-sectional survey of knowledge, attitudes and practices around COVID-19 among individuals in 23 countries and territories (Table 1). The survey was conducted by UNICEF (Middle East and North Africa Region) and WHO (Eastern Mediterranean Region).

**Table 1:** Participants recruited to the survey of COVID-19 vaccination status and adherence to preventive measures, Eastern Mediterranean Region and Algeria, June to November 2021

Country or territory, by income group	No. of participants	
	Round 1: Jun–Jul 2021	Round 2: Oct–Nov 2021
**High income **		
Bahrain	354	350
Kuwait	501	511
Oman	500	503
Qatar	350	352
Saudi Arabia	761	755
United Arab Emirates	502	500
**Middle income**		
Algeria	706	700
Djibouti	350	350
Egypt	1001	1059
Iran (Islamic Republic of)	1002	1030
Iraq	793	716
Jordan	552	520
Lebanon	500	504
Libya	520	511
Morocco	715	772
Pakistan	1026	1016
Tunisia	575	628
West Bank and Gaza Strip	359	350
**Low income** ** **		
Afghanistan	775	713
Somalia	501	507
Sudan	769	761
Syrian Arab Republic	634	523
Yemen	541	500
**Total**	**14 287**	**14 131**

COVID-19: coronavirus disease 2019.

Note: Country groups are those of the 2021 World Bank income classification.^19^

Source: United Nations Children’s Fund (Middle East and North Africa Region) and World Health Organization (Eastern Mediterranean Region) survey of COVID-19 knowledge, attitudes and practices.

### Data collection

We conducted the survey in two waves, first in June and July 2021 and then in October and November 2021. We based the survey on computer-assisted telephone interviews, using random digital dialling to sample working mobile numbers in each country. We hired a service provider company (GeoPoll, Denver, United States of America) to conduct the survey.

For the data collection we designed a structured questionnaire consisting of 31 standardized questions related to: (i) individual characteristics, such as demographic, employment, health and vaccination status; (ii) behavioural barriers, perceptions and beliefs about vaccines and COVID-19; and (iii) community factors, such as social norms and impact on households and health service utilization. We derived the questions from the global question bank provided by the Risk Communication and Community Engagement Collective Service, a collaborative partnership of key stakeholders from the public health and humanitarian sectors. The choice of questions was guided by a conceptual model of vaccine uptake.^20^ The model is well-recognized and embraces an ecological approach by including individual and community influences as well as wider policy and environmental factors and influences on health decisions (the model is shown in the online repository).^21^ The questionnaire was translated into national languages. We piloted the survey in all countries before starting data collection.

For the full data collection, trained enumerators used random digit dialling to generate a random sample of mobile phone respondents aged 18 years and older from the 22 countries and one territory. The analysis comprised separate samples of 14 287 individuals in the first round and 14 131 individuals in the second round. Sample sizes for each country were based on population size and mobile phone coverage. Participants’ details were anonymized after each round of data collection; it was therefore not possible to determine the overlap of participants in the first and the second round of the survey. However, even in the least populous countries the chances of selecting the same number with random digit dialling is very small. For example, Djibouti has a population of 1.1 million and we selected around 400 participants. Based on the proportion of adults in the population (63%) and the mobile phone coverage (43%), there are still around 300 000 eligible respondents in the country. We weighted the sample at regional level by gender and age, based on the United Nations (UN) demographics for the countries and territory.^22^ A detailed description of the derivation of the weights is presented in the online repository.^21^

### Statistical analysis

The variables concerning adherence to public health and social measures were based on responses to the questions about the frequency of practising the following measures over the previous week: (i) wearing a mask in public; (ii) keeping a physical distance of at least 2 m from people in public; and (iii) washing hands with soap and water for 20 seconds. Respondents gave their responses on a 5-point Likert scale (1: all of the time; 5: never). We defined binary outcome variables for each of the three public health and social measures, taking a value of 1 if the respondent practised the measure all of the time or most of the time and 0 for sometimes, rarely or never. The variable concerning vaccination status was based on participants’ responses to the question about whether they had received at least one dose of COVID-19 vaccine. From the responses we created a binary variable with values of 1 if the respondent had been vaccinated and 0 if not vaccinated.

To study the correlates of each of the public health and social measures we developed three separate models. The binary variables for practice of public health and social measures were then used as dependent variables in a logit modelling analysis. In addition to the variable capturing vaccination status, the model included the following correlates: socioeconomic and demographic variables (such as age, gender, occupation); self-reported previous infection with severe acute respiratory syndrome coronavirus 2 (SARS-CoV-2); and knowledge about asymptomatic SARS-CoV-2 transmission (including attitudes towards being at risk). By asking about respondents’ risk beliefs, we were able to capture infection risk as well as self-reported vaccination status. 

To account for country heterogeneity (for example, differences in the health-care systems), we included country dummy variables in the regression analysis. All regressions used the derived weights as described above (further details are in the online repository).^21^ We conducted the analysis on the first round of collected data and then repeated it on the second round of data. Given that our sample included respondents living in countries at different levels of economic development, we also analysed the countries grouped by the World Bank categories: high, middle and low income.^19^

We performed three additional analyses. First, we fitted our model onto a pooled data set comprising the two rounds of the survey, while also accounting for the time effect as well as an interaction variable between the time effect and the vaccination status variable. With this method we aimed to test if the link between vaccination status and adherence to preventive measures changed over time. Second, we conducted a propensity score matching analysis, which is an established technique to reduce selection bias in observational data, by matching treatment and control individuals (that is, vaccinated and non-vaccinated individuals) based on their observable characteristics.^23^^,^^24^ In doing so, we compared the practice of preventive measures of those respondents who were vaccinated (treated) with those who were not vaccinated (control group). In particular, we used a nearest neighbour matching estimator. A treatment group observation (vaccinated individual) thus matched with an observation from the control group (unvaccinated individual) that had the closest propensity score^25^^,^^26^ (further details are in the online repository).^21^ Finally, we conducted a multinomial logit analysis using untransformed public health and social measures variables (as categorical variables on a 5-point Likert scale).

We performed all analyses using Stata version 14.0 (StataCorp., College Station, USA); *P*-values less than 0.05 were considered significant throughout.

### Ethical approval 

The survey tool and protocols were approved by the WHO Regional Ethical Research Committee.

## Results

### Background characteristics

The samples in round 1 and round 2 were similar in terms of the distribution of age and gender (Table 2). Understandably, the numbers of respondents reporting that they had ever been infected with COVID-19 increased from 1950 in the first round to 2637 in the second round (weighted percentage of total respondents 13.9% and 19.0%, respectively). The number of respondents reporting that they were vaccinated increased from 2772 to 8766 between rounds (19.4% to 62.3%; weighted percentages). Finally, while the practice of physical distancing and handwashing was similar between the two rounds of the survey, the number of respondents reporting wearing a mask in public decreased from 9370 to 8674 (66.0% to 61.6%; weighted percentages). Similar findings emerged when the descriptive statistics were analysed by country income groups (available in the online repository).^21^ More specifically, the substantial increase in self-reported vaccination status was accompanied by only a small decrease in adherence to public health and social measures.

**Table 2:** Characteristics of respondents in the survey of COVID-19 vaccination status and adherence to preventive measures, Eastern Mediterranean Region and Algeria, June to November 2021

Variable	No. of respondents (weighted %)	
	Round 1: Jun–Jul 2021 (*n* = 14 287)	Round 2: Oct–Nov 2021 (*n* = 14 131)
**Gender and age**		
Female		
Age 18–24 years	1 432 (10.0)	1 416 (10.0)
Age 25–34 years	1 810 (12.7)	1 790 (12.7)
Age 35–49 years	1 986 (13.9)	1 964 (13.9)
Age 50+ years	1 678 (11.7)	1 659 (11.7)
Male		
Age 18–24 years	1 521 (10.6)	1 505 (10.6)
Age 25–34 years	1 982 (13.9)	1 960 (13.9)
Age 35–49 years	2 172 (15.2)	2 149 (15.2)
Age 50+ years	1 707 (11.9)	1 688 (11.9)
**Occupation**		
Working in education sector	599 (4.2)	780 (5.5)
Working in health-care sector	357 (2.5)	623 (4.4)
Homemaker	2 437 (17.2)	3 050 (21.7)
Not currently in paid work	2 689 (19.0)	2 619 (18.6)
Working in other essential services^a^	6 684 (47.3)	5 602 (39.8)
Student	1 368 (9.7)	1 382 (9.8)
**Do you have a chronic illness?**		
Yes	2 276 (16.0)	2 432 (17.3)
No	11 945 (84.0)	11 655 (82.7)
**To your knowledge, are you or have you been infected with COVID-19? **		
Yes	1 950 (13.9)	2 637 (19.0)
No	12 042 (86.1)	11 270 (81.0)
**Have you received COVID-19 vaccination?**		
Yes	2 772 (19.4)	8 766 (62.3)
No	11 500 (80.6)	5 297 (37.7)
**To what extent do you trust your local health-care providers to provide accurate information on COVID-19 vaccination and prevention?**		
Extremely	1 430 (15.0)	2 108 (19.4)
Very much	2 510 (26.2)	3 409 (31.4)
Moderately	2 837 (29.7)	2 931 (27.0)
Slightly	1 428 (14.9)	1 550 (14.3)
Not at all	940 (9.8)	851 (7.8)
**Do you believe coronavirus can be transmitted by coming in direct contact with a person who has the virus but has no symptoms?**		
Yes	7 242 (75.7)	7 349 (70.7)
No	1 654 (17.3)	3 051 (29.3)
**How likely do you believe that you will get infected with COVID-19?**		
Very likely	1 029 (10.8)	915 (8.9)
Likely	2 260 (23.6)	2 181 (21.3)
Neutral	1 647 (17.2)	1 839 (18.0)
Unlikely	1 344 (14.1)	1 802 (17.6)
Very unlikely	2 432 (25.4)	3 504 (34.2)
**Over the past week how often have you:**		
Worn a mask in public	9 370 (66.0)	8 674 (61.6)
Kept at least 2 m away from people in public	7 286 (51.7)	7 046 (50.2)
Washed your hands with water and soap for 20 seconds	11 116 (78.3)	10 729 (76.3)

COVID-19: coronavirus disease 2019.

^a^ Other services deemed essential during the pandemic, such as food sector, logistic.

Note: *n* is the total sample size in each round. We calculated percentages by applying the appropriate regional weights; data were missing in some categories. Inconsistencies arise in some values due to rounding. All variables were self-reported.

Source: United Nations Children’s Fund (Middle East and North Africa Region) and World Health Organization (Eastern Mediterranean Region) survey of COVID-19 knowledge, attitudes and practices.

### Logit model analysis

**Fig. 1:**
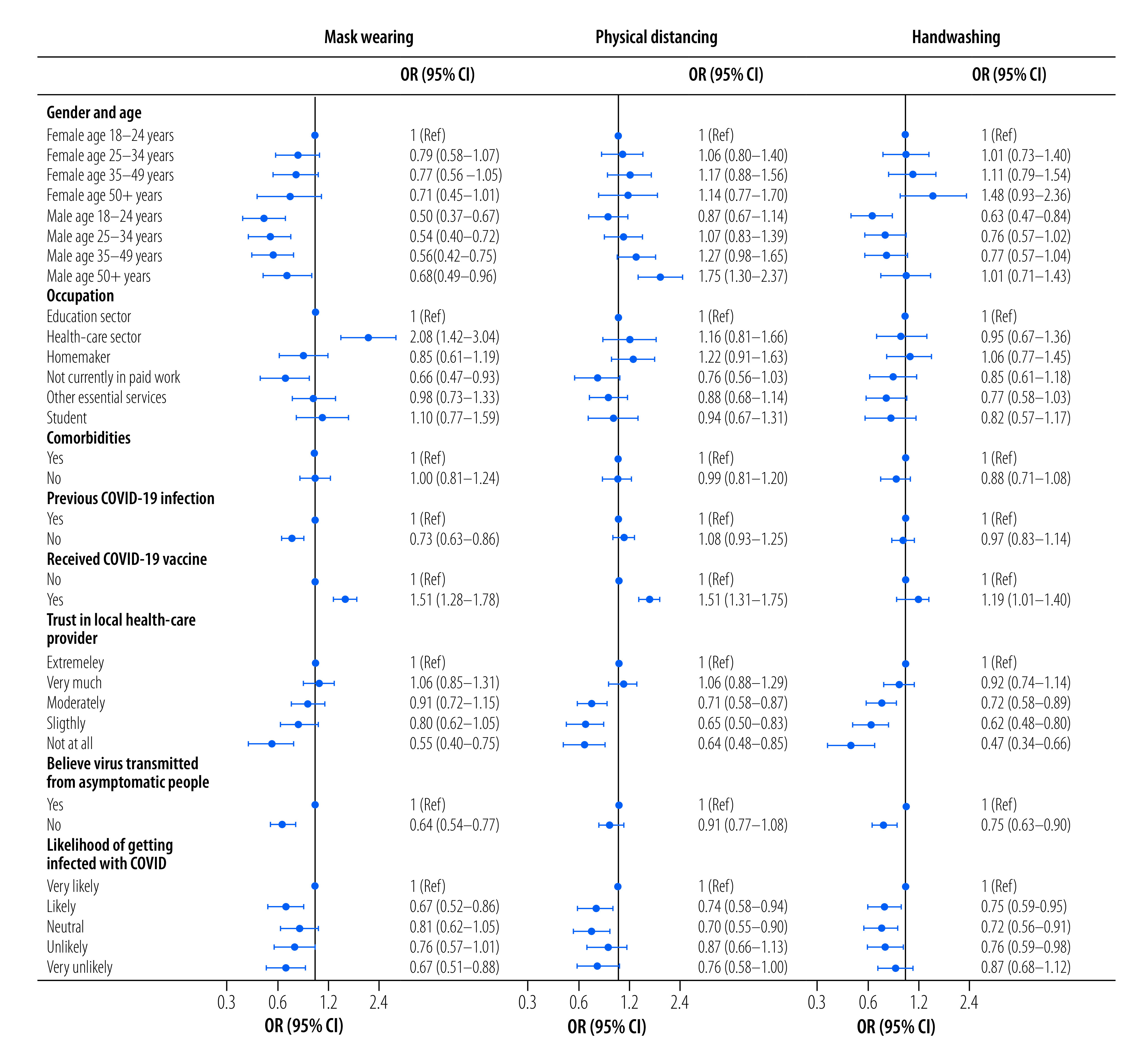
Likelihood of adhering to preventive measures, Eastern Mediterranean Region and Algeria, October to November 2021

CI: confidence interval; COVID-19: coronavirus disease 2019; OR: odds ratio; Ref: reference group.

Note: All variables were self-reported.

Source: Round 2 (October and November 2021; *n* = 14 131 participants) of the United Nations Children’s Fund (Middle East and North Africa Region) and World Health Organization (Eastern Mediterranean Region) survey of COVID-19 knowledge, attitudes and practices.

Fig. 1 depicts the findings of the logit model analysis based on data of round 2 of the survey. Demographic status played a significant role in mask wearing, with young men being less likely relative to young women to wear a mask. More specifically, men aged 18–24 years were 0.5 times less likely (95% confidence interval, CI: 0.4–0.7), and men aged 25–34 years were 0.5 times less likely (95% CI: 0.4–0.7) to wear a mask in public compared with women aged 18–24 years. In addition, those reporting no previous COVID-19 infection were 0.7 times less likely (95% CI: 0.6–0.9) to wear a mask in public. Respondents who did not know that the SARS-CoV-2 could be passed on asymptomatically were 0.6 times less likely (95% CI: 0.5–0.8) to report wearing a mask in public. More importantly, the results show a strong link between self-reported COVID-19 vaccination status and wearing a mask in public. Those who had been vaccinated were 1.5 times more likely (95% CI: 1.3–1.8) to report wearing a mask in public relative to those who were not vaccinated. 

When considering physical distancing, we found some evidence that those with lower trust in the local health-care provider were less likely to practise physical distancing (Fig. 1). However, there is a strong link between vaccination status and physical distancing, suggesting that those who were vaccinated were 1.5 times more likely (95% CI: 1.3–1.7) to practise physical distancing relative to unvaccinated respondents. 

Finally, when using handwashing with water and soap for 20 seconds as a dependent variable, younger men, those with lower trust in the local health-care provider, as well as those who did not consider themselves to be at risk of contracting SARS-CoV-2, were less likely to adhere to frequent handwashing. We also found that COVID-19 vaccinated respondents were 1.2 times more likely (95% CI: 1.0–1.4) to wash their hands regularly than were unvaccinated respondents. These findings were similar when the analysis was repeated on data from the round 1 survey (available in online repository).^21^

### Income group analysis

**Fig. 2:**
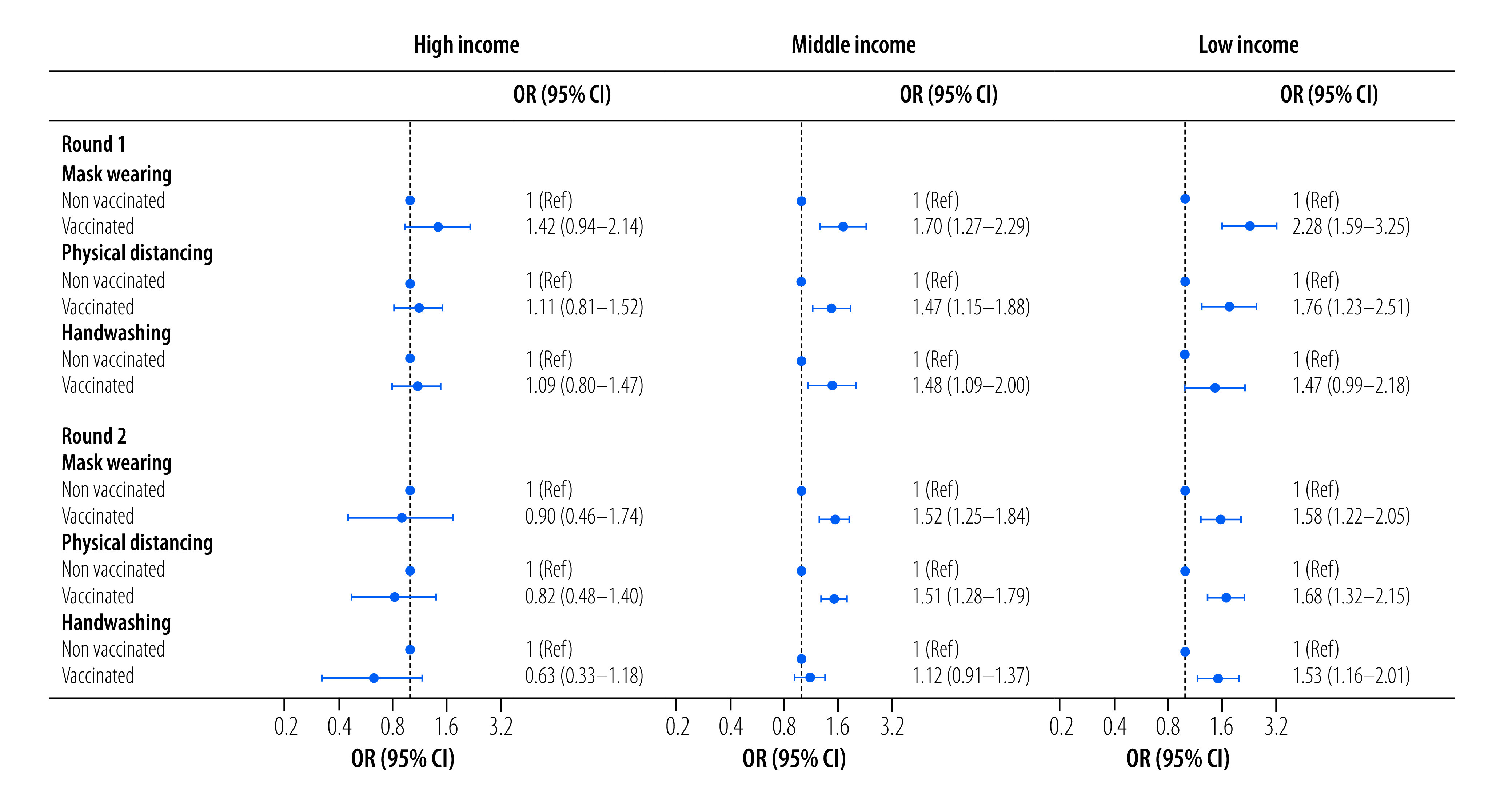
Likelihood of adhering to preventive measures, by vaccination status and country income group, Eastern Mediterranean Region and Algeria, June to November 2021

CI: confidence interval; COVID-19: coronavirus disease 2019; OR: odds ratio; Ref: reference group.

Notes: The models above control for the following set of correlates: age, gender, occupation status, existence of comorbidities, previous COVID-19 infection, trust in local health-care provider, knowledge about asymptomatic transmission of the virus, risk attitude towards COVID-19. Country groups are those of the 2021 World Bank income classification (Table 1). All variables were self-reported.

Source: Round 1 (June and July 2021; *n* = 14 287 participants) and round 2 (October and November 2021; *n* = 14 131 participants) of the United Nations Children’s Fund (Middle East and North Africa Region) and World Health Organization (Eastern Mediterranean Region) survey of COVID-19 knowledge, attitudes and practices.

Given the income heterogeneity of our sample, we next conducted a subregional analysis for high-, middle- and low-income countries. Fig. 2 illustrates the odds ratios of the logit model for our main variable of interest (vaccination status), while the logit models also controlled for the same set of variables used in Fig. 1 above. There was no statistically significant link between COVID-19 vaccination status and adherence to public health and social measures in the high-income countries. However, in the middle-income countries and territory there was a positive link between vaccination and practice of preventive measures, except for handwashing in round 2. In addition, the odds ratios for adherence to some of the public health and social measures were comparable across rounds. For example, in both rounds, those vaccinated were 1.5 times more likely to physically distance than those who were unvaccinated. These findings were similar when the analysis was repeated on the subsample of low-income countries (Fig. 2).

### Additional analyses

Detailed results of all additional analyses are available in the online repository.^21^ As a robustness check, we repeated the analysis by pooling the two rounds of the survey. In addition, the time effect was statistically significant across all three public health and social measures and with odds ratios lower than 1, indicating, on average, a reduction in the practice of the selected preventive measures over time. Finally, in the case of mask wearing and physical distancing, the interaction term between the time effect and self-reported vaccination status was insignificant, suggesting that the link between vaccination and preventive measures had not changed over time. These results are consistent when the analysis was repeated on the subregional level by country income group.

Furthermore, when we used propensity score matching in the analysis, the results confirmed our main findings. Finally, the results were also similar when we used a multinomial logit analysis on untransformed health practice variables in the analysis.

## Discussion

We found a robust link between self-reported COVID-19 vaccination status and adherence to all three public health and social measures in the Eastern Mediterranean Region and Algeria. Individuals vaccinated against COVID-19 were more likely to adhere to preventive behaviours compared with unvaccinated respondents in low- and middle-income countries and territory. This finding supports the general health motivation construct in the health belief model,^27^ and aligns with social identity theory.^28^ The theory proposes that people who practise one health behaviour (such as vaccination) are more likely to practise others (in this case, recommended measures to prevent the transmission of SARS-CoV-2). Furthermore, it may also be the case that, after receiving the vaccine at their vaccination appointment, individuals are reminded to continue their practice of mask wearing, physical distancing and handwashing.^29^ However, we did not find a statistically significant link between vaccination status and adherence to preventive measures in the high-income countries. The countries in the Gulf have been able to achieve a more rapid roll-out of COVID-19 vaccination programmes than low- and middle-income countries, resulting in a substantial increase in vaccination coverage in a relatively short period. According to official data, by the end of 2021 about three quarters of the adult population in the Gulf countries had received at least one dose of a COVID-19 vaccine.^30^ This high coverage may explain the lack of a statistically significant link between vaccination status and adherence to public health and social measures. Similar results have been reported from other advanced economies across the world.^4^^,^^5^^,^^31^

Nevertheless, the results of the pooled analysis suggest that adherence to preventive measures decreased slightly over the 5 months between surveys, which is somewhat consistent with people losing motivation to follow recommended protective measures.^3^ However, our analysis also indicates that, except for handwashing, the link between vaccination and the practice of preventive measures did not change between the two survey rounds, despite the surge in vaccination coverage in the region. The results are robust when we used alternative methods of analysis (propensity score matching and multinomial logit). Finally, we also found evidence that demographic characteristics, trust in the local health-care provider, as well as risk perception of COVID-19, were significant correlates of individuals’ adherence to preventive measures. Previously, it has been shown that declining prevalence and severity of COVID-19 was associated with lower adherence to preventive measures in Australia, the United Kingdom and the USA,^32^ as well as Somalia.^7^

We also found consistent evidence that demographic factors, trust in local health-care providers, as well as perception of COVID-19 risk, are significant correlates of adherence to preventive measures. Studies in the same region have shown that women and older individuals were more adherent to COVID-19 preventive measures.^9^^,^^11^^,^^15^^,^^17^ In addition, existing studies from the region indicated that lower trust in health-care providers was associated with lower adherence to preventive behaviours.^13^ Finally, individuals who believed they were at lower risk of contracting COVID-19 tended to show lower adherence to public health and social measures, consistent with existing evidence.^33^ It is worth pointing out that the risk of infection was different in the two rounds of the survey, in that lockdowns and other policies had reduced the risk of infection over time.

Our study has several limitations. The first limitation is in the study’s representativeness. People who did not have mobile phones or chose not to participate were not included in the study (although in 2020, the average mobile phone penetration in the region was 98 mobile phone subscriptions per 100 people).^34^ In addition, and given the cultural traditions of the region, more men than women tended to be included, using the sampling method applied here. To mitigate this bias, we used UN standard population demographic data to weight the raw data by age and gender to adjust for such differences. Second, the analysis in our study was based on self-reported data, which is vulnerable to various types of bias. We explored this bias through comparison with other data sources, such as vaccination status. Third, the survey was a repeated cross-sectional survey, so we caution against direct causal inferences, particularly as we gathered data on vaccination status and practice of preventive measures at the same time. Fourth, we only conducted regional and subregional analyses, as the sample was not stratified by subnational or administrative level, and country samples were too small to support individual analysis. Finally, public opinion about the risks of COVID-19 is dynamic. These data were collected during June and July and then October and November 2021. Interpretation of the data should consider factors affecting countries around that time, such as government policies and enforcement of restrictions, seasonal activities, traditions or conventions related to education calendars, cultural and religious events, and the media.

Overall, in the regions we studied, we found no evidence that the roll-out of vaccination programmes resulted in COVID-19 risk compensation, whereby individuals adjust their behaviour based on a lower perceived level of risk. Nevertheless, reinforcement of public health messages on prevention is still important for individuals’ compliance and, in an era when government-mandated restrictions are being lifted, adherence to public health and social measures are expected to drop. 
